# The Navajo Nation Healthy Diné Nation Act: A Two Percent Tax on Foods of Minimal-to-No Nutritious Value, 2015–2019

**DOI:** 10.5888/pcd17.200038

**Published:** 2020-09-03

**Authors:** Del Yazzie, Kristen Tallis, Caleigh Curley, Priscilla R. Sanderson, Regina Eddie, Timothy K. Behrens, Ramona Antone-Nez, Martin Ashley, Herbert John Benally, Gloria Ann Begay, Shirleen Jumbo-Rintila, MA, Hendrik D. de Heer

**Affiliations:** 1 Navajo Department of Health, Navajo Epidemiology Center, Window Rock, Arizona; 2 Northern Arizona University, Department of Health Sciences, Flagstaff, Arizona; 3 Northern Arizona University, School of Nursing, Flagstaff, Arizona; 4 University of Wisconsin-Milwaukee, College of Health Sciences, Milwaukee, Wisconsin; 5 Office of the Navajo Nation Tax Commission, St. Michaels, Arizona; 6 Diné College, School of Diné Studies and Education, Shiprock, Arizona; 7 Diné Community Advocacy Alliance, Church Rock, New Mexico; 8 Navajo Division of Community Development, Window Rock, Arizona

## Abstract

Our study summarizes tax revenue and disbursements from the Navajo Nation Healthy Diné Nation Act of 2014, which included a 2% tax on foods of minimal-to-no nutritional value (junk food tax), the first in the United States and in any sovereign tribal nation. Since the tax was implemented in 2015, its gross revenue has been $7.58 million, including $1,887,323 in 2016, the first full year. Revenue decreased in absolute value by 3.2% in 2017, 1.2% in 2018, and 4.6% in 2019, a significant downward trend (*P* = .02). Revenue allocated for wellness projects averaged $13,171 annually for each local community, with over 99% successfully disbursed and more rural areas generating significantly less revenue. Our results provide context on expected revenue, decreases over time, and feasibility for tribal and rural communities considering similar policies.

**Summary**

**What is already known on this topic?**

Food and beverage taxation may be a cost-effective public health strategy, but no unhealthy food tax has been implemented in the United States or among a tribal nation at high risk for chronic conditions.

**What is added by this report?**

The Healthy Diné Nation Act, a 2% tax on unhealthy foods, generated over $13,000 annually per Navajo rural community for local wellness programming, of which 99.1% was disbursed. An average 3% revenue decrease was recorded each year with no retail-related sales tax decline.

**What are the implications for public health practice?**

Successful disbursements suggest feasibility of unhealthy foods policies in a sovereign tribal nation that serves an indigenous population at high risk for common chronic conditions.

## Objective

To promote the health of the Navajo people, the Navajo Tribal Council passed the Healthy Diné Nation Act (HDNA) (1) in November 2014, which enacted a 2% tax on unhealthy or minimal-to-no nutritional value foods (junk foods). The HDNA law defines these foods as sweetened beverages, prepackaged and nonprepackaged snacks stripped of essential nutrients and high in salt, saturated fat, and sugar, including sweetened beverages, sweets, and chips and crisps. Aligned with tribal government structures, HDNA tax revenues were allocated for disbursement directly to each of 110 Navajo Nation communities, also called chapters, with an average of about 1,600 residents (2). Funds were disbursed for local wellness programming such as farming, traditional food demonstrations, exercise equipment, walking trails, and community cleanup (1).

Although the HDNA is the first unhealthy food tax in the United States, there are examples of similar taxes (3–8) internationally and in several large municipalities in the United States (9,10). Those taxes were typically higher than the 2% HDNA tax, and consumption and revenue decreased after enactments. Studies examining enactments found stronger effects among low-income households (3,4,7) and in urban areas (vs rural) (6–8). However, to date, no study has documented the impact of this type of policy among a tribal or indigenous nation at high risk for chronic diseases such as type 2 diabetes.

Our aim of this report is to summarize the HDNA tax revenue over time, with variation by region, and disbursement of revenue to rural communities for wellness projects. Because the 2% tax was much lower than similar policies shown to impact purchasing behaviors in other countries, we hypothesized that HDNA revenue would not change substantially over time and that revenue would be lowest in the most rural areas.

## Methods

HDNA tax revenue and sales tax data were provided by the Office of the Navajo Tax Commission and summarized for 17 consecutive quarters, starting July to September 2015. Gross tax revenue was first calculated. Next, funds were allocated for disbursement to the 110 local chapters for wellness projects. Disbursements for chapters (gross revenue minus 20% set asides [eg, Veterans Trust Fund]) were summarized across 5 regional agencies by using a 50-50 formula; that is, 50% of tax revenue collected within an agency was evenly distributed to chapters within that agency, and the remaining 50% was distributed based on voter registration enrollment. Disbursement data were collected from the Navajo Division of Community Development and cross-checked with tax commission totals.

Microsoft Excel (Microsoft) and SPSS v.26.0 (IBM) were used to characterize data by using descriptive statistics. One-way analysis of variance (ANOVA) with post hoc comparisons and a Bonferroni correction to adjust for multiple comparisons were used to test whether revenue (absolute and adjusted for inflation) and disbursements differed by quarter and between regional agencies. Linear regression analysis tested whether a significant trend occurred in total revenue. Visual displays were created by using ArcMap v.10.7.1 (Esri). The project was approved by the Navajo Nation Human Research Review Board (NNR.17–284T).

## Results

### Gross revenue

**Figure 1:**
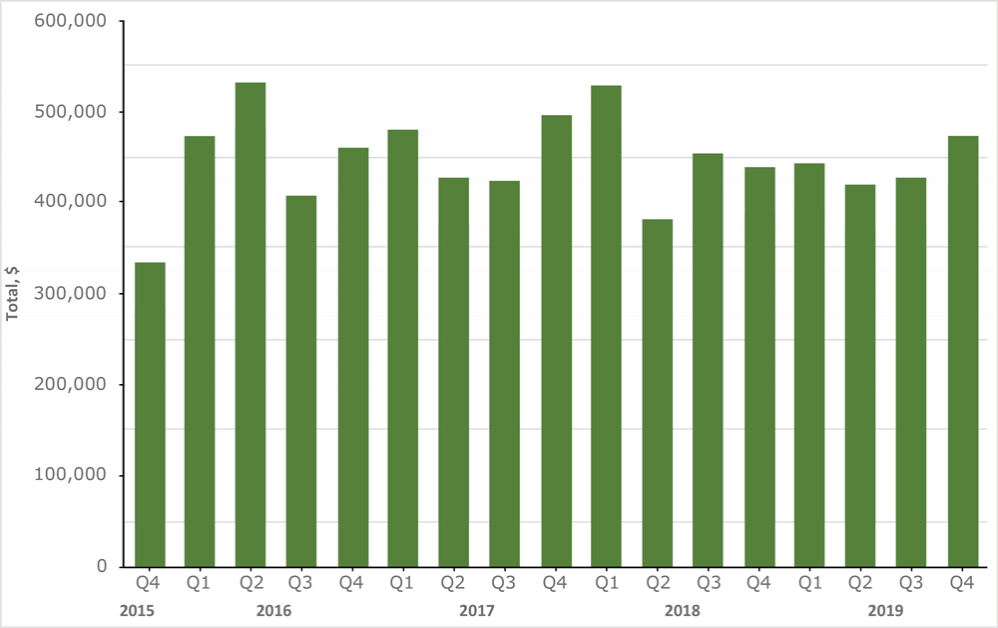
Navajo Nation Healthy Diné Nation Act tax revenue on foods of minimal-to-no nutritious value by quarter, 2015–2019. The chart shows revenue by quarter for each fiscal year. Data range from the final quarter of 2015 through the final quarter of 2019. Revenue was the lowest in the first quarter of collections with just over $334,000, and the highest in the second quarter of 2016 with $533,417.

From 2015 through 2019, total revenue was $7.58 million. Average revenue was highest ($481,425) in quarter 1, October to December, and lowest in quarter 3, April to June ($426,147), although one-way ANOVA post hoc comparisons did not reveal significant differences between quarters (*P* = .48). For 2016, the first full year, revenue was $1,887,323, which decreased in absolute value by 3.2% in 2017, another 1.2% in 2018, and another 4.6% in 2019 (Figure 1). Linear regression indicated a significant downward trend of absolute revenue across all years [*F* = 41.23; degrees of freedom (df) = 3; β = −$51,675; *P* = .02]. Adjusted for food cost inflation in the United States during the same time, revenue decreased 12.4% from 2016 through 2019, or 4.1% per year (*F* = 68.92; df = 3; β = −$78,033; *P* = .014). Revenues represent a total of $379 million ($90 million/y) in taxed unhealthy food purchases since implementation. Despite waiving sales tax on fruits, vegetables, water, and nuts in 2015, sales tax did not decrease over that period. Total retail-related sales tax revenue was $10,320,171 in 2015 and $10,846,203 in 2018 ($10,237,606 inflation-adjusted), which was not significantly different.

**Table:** 

Year	Total
**2015**	
Quarter 4	334,084
**2016**	
Quarter 1	473,851
Quarter 2	533,417
Quarter 3	414,613
Quarter 4	465,442
**2017**	
Quarter 1	477,930
Quarter 2	426,500
Quarter 3	424,618
Quarter 4	498,963
**2018**	
Quarter 1	531,225
Quarter 2	378,052
Quarter 3	454,448
Quarter 4	442,412
**2019**	
Quarter 1	442,696
Quarter 2	396,370
Quarter 3	410,912
Quarter 4	472,386

### Distribution and geographic variation

Disbursements occurred each year after collection for a total of $6,062,335 for local community wellness projects over the 4 years. This averages $55,112 per chapter or $13,171 per year. Specifically, $13,726 was disbursed for collections in 2015, $13,295 for 2016, $13,136 for 2017, and $12,527 for 2018. From 2015 through 2019, 435 disbursements were made from a possible 440. In 2016, 107 of 110 communities received full funds, and all 110 communities received full funds by 2018. To date, $4,641,935 of $4,684,443 (99.1%) of revenue funds were disbursed.

**Figure 2:**
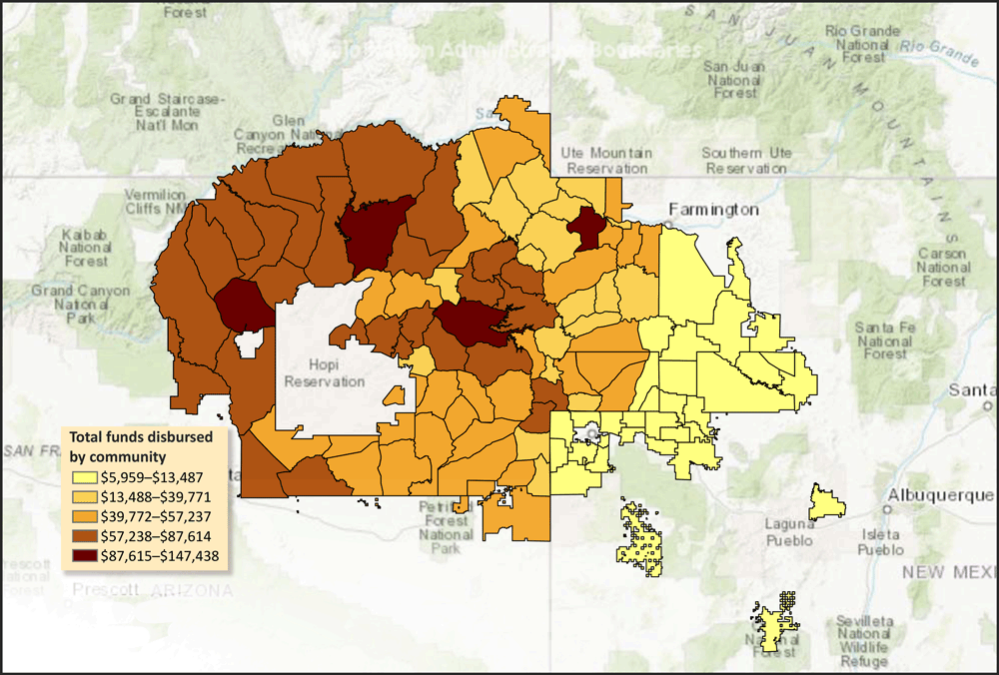
Map of Navajo Nation administrative boundaries shows funds disbursed from the Navajo Nation Healthy Diné Nation Act tax on foods of minimal-to-no nutritious value to each Navajo Nation community, 2016–2019 (11,12). Sources: Administrative boundary data from the Navajo Abandoned Uranium Mines project. The map includes Agencies, Districts, Chapters, and Abandoned Uranium Mine Regions of the Navajo Nation. Outlying areas are compiled from several data providers, including the US Geological Survey, US Environmental Protection Agency, US National Park Service, Food and Agriculture Organization of the United Nations, Department of Natural Resources Canada, Geo Base, Agriculture and Agri-Food Canada, Garmin, HERE, Esri, OpenStreetMap contributors, and the GIS User Community. For more information on this map, visit http://goto.arcgisonline.com/maps/World_Topo_Map.

Disbursements varied by regions within the Navajo Nation. Four of 5 regional agencies generated at least $1 million in revenue through 2018. From 11 possible agency comparisons, 9 were significantly different with 8 *P* values of less than .001. Although the Navajo Nation is considered rural, the Eastern agency had the smallest average community size of 1,074 residents and had a lower revenue than all other agencies (*P* < .001). Western Navajo agency generated the most revenue ($1,732,949) and had the largest average community size of an estimated 2,090 residents (Figure 2).

## Discussion

The Navajo Nation 2% tax on foods of minimal-to-no nutritious value has generated $7.58 million since late 2015. This means that about $13,000 was available each year for wellness projects for 110 communities of about 1,600 residents, with over 99% of available funds disbursed. These findings suggest feasibility of the Healthy Diné Nation Act (HDNA) policy in terms of collection and disbursement aligned with tribal governance.

Revenue decreased an average 3% per year (4% when adjusted for inflation), a significant downward trend. Although much higher than the HDNA 2% tax, unhealthy food taxes in other settings reduced consumption, ranging from a 3.4% to 5.1% decrease in unhealthy food purchases in Hungary (4) and Mexico (5) to more than 20% lower sugar-sweetened beverage purchases in Berkeley, California (9), and Philadelphia, Pennsylvania (14). Effects in other studies varied by income. In Mexico, high-income households did not change unhealthy food consumption, whereas low-income households decreased consumption by over 10% (5). We found some evidence of a downward trend in HDNA tax revenue, without declines in retail-related sales tax revenue. Perhaps the combination of the tax itself, a population with high poverty rates (13), and the funding of local wellness projects with the tax revenue might have increased awareness of the policy and healthy eating, resulting in modest reductions in purchasing behaviors.

The public health implications of the current work are that a 2% unhealthy food tax in a rural, tribal area generated about $13,000 per rural community per year, that modest reductions of 1% to 4% annually can be expected, and that distributing funds to small local communities for wellness projects appears feasible within tribal government structures. Future research will use a Navajo-specific Behavioral Risk Factor Surveillance Survey (13) to gain insight into whether revenue decreases can be explained by reductions in consumption of unhealthy foods or other factors, such as changes in the food store environment. Our research has particular relevance for other sovereign indigenous nations and rural communities considering similar policies and funding structures in an effort to meaningfully engage their populations in chronic disease prevention (14). More research is urgently needed, particularly to inform the HDNA law renewal.
